# Supplementary material for: Hybrid Models and Biological Model Reduction with PyDSTool
Source: PLoS Comput Biol. 2012 Aug 9;8(8):e1002628. doi: 10.1371/journal.pcbi.1002628 (PMC3415397; doi:10.1371/journal.pcbi.1002628)
Supplement: Text S4 — Complete source code for the PyDSTool package (version 0.88.120504). Includes API documentation and help files linking to web pages. This file is identical to the current public release on Sourceforge.net. (ZIP) [file pcbi.1002628.s004.zip › PyDSTool/html/PyDSTool.Toolbox.optimizers.criterion.composite_criteria-pysrc.html]

xml version="1.0" encoding="ascii"?


PyDSTool.Toolbox.optimizers.criterion.composite\_criteria


| Home | Trees | Indices | Help | PyDSTool |
| --- | --- | --- | --- | --- |

Package PyDSTool ::
Package Toolbox ::
Package optimizers ::
Package criterion ::
Module composite\_criteria

[hide private]

[frames] | no frames]

# Source Code for Module PyDSTool.Toolbox.optimizers.criterion.composite\_criteria

```
 1  
 
 2  # Matthieu Brucher
 
 3  # Last Change : 2007-08-24 10:25
 
 4  
 
 5  """
 
 6  Composite criteria allow to use several criteria together, with and/or composition
 
 7  """ 
 8  
 
 9  import sys 
10  
 
11  if sys.version < '2.5': 


12 -  def all(iterable):


13      for element in iterable: 
14          if not element: 
15              return False 
16      return True

17  
 


18 -  def any(iterable):


19        for element in iterable: 
20            if element: 
21                return True 
22        return False

23  
 


24 -class OrComposition(object):


25    """
 
26    Compose several criteria with an or rule
 
27    """ 


28 -  def __init__(self, *args, **kwargs):


29      """
 
30      Collects the different criteria
 
31      """ 
32      self.criteria = kwargs.values() + list(args)

33  
 


34 -  def __call__(self, state, **kwargs):


35      """
 
36      Evaluates each criterion (no lazy evaluation) and returns True if one of them is True
 
37      """ 
38      r = [criterion(state, **kwargs) for criterion in self.criteria] 
39      return any(r)

40  
 


41 -class AndComposition(object):


42    """
 
43    Compose several criteria with an and rule
 
44    """ 


45 -  def __init__(self, *args, **kwargs):


46      """
 
47      Collects the different criteria
 
48      """ 
49      self.criteria = kwargs.values() + list(args)

50  
 


51 -  def __call__(self, state, **kwargs):


52      """
 
53      Evaluates each criterion (no lazy evaluation) and returns True if one of them is True
 
54      """ 
55      r = [criterion(state, **kwargs) for criterion in self.criteria] 
56      return all(r)

57
```

  


| Home | Trees | Indices | Help | PyDSTool |
| --- | --- | --- | --- | --- |

|  |  |
| --- | --- |
| Generated by Epydoc 3.0.1 on Fri May 4 15:24:14 2012 | http://epydoc.sourceforge.net |
